# CCL2 upregulation triggers hypoxic preconditioning-induced protection from stroke

**DOI:** 10.1186/1742-2094-9-33

**Published:** 2012-02-16

**Authors:** Ann M Stowe, Bradley K Wacker, Petra D Cravens, Jennifer L Perfater, Min K Li, Ruilong Hu, Angela B Freie, Olaf Stüve, Jeffrey M Gidday

**Affiliations:** 1 Department of Neurological Surgery, Washington University School of Medicine, 660 S. Euclid Ave., Box 8057, St. Louis, MO 63110, USA; 2 Cell Biology & Physiology, Washington University School of Medicine, St. Louis, MO, USA; 3 Department of Neurology and Neurotherapeutics, UT Southwestern Medical Center, Dallas, TX, USA; 4 Neurology Section, VA North Texas Health Care Systems, Dallas, TX, USA

## Abstract

**Background:**

A brief exposure to systemic hypoxia (i.e., hypoxic preconditioning; HPC) prior to transient middle cerebral artery occlusion (tMCAo) reduces infarct volume, blood-brain barrier disruption, and leukocyte migration. CCL2 (MCP-1), typically regarded as a leukocyte-derived pro-inflammatory chemokine, can also be directly upregulated by hypoxia-induced transcription. We hypothesized that such a hypoxia-induced upregulation of CCL2 is required for HPC-induced ischemic tolerance.

**Methods:**

Adult male SW/ND4, CCL2-null, and wild-type mice were used in these studies. Cortical CCL2/CCR2 message, protein, and cell-type specific immunoreactivity were determined following HPC (4 h, 8% O_2_) or room air control (21% O_2_) from 6 h through 2 weeks following HPC. Circulating leukocyte subsets were determined by multi-parameter flow cytometry in naïve mice and 12 h after HPC. CCL2-null and wild-type mice were exposed to HPC 2 days prior to tMCAo, with immunoneutralization of CCL2 during HPC achieved by a monoclonal CCL2 antibody.

**Results:**

Cortical CCL2 mRNA and protein expression peaked at 12 h after HPC (both *p *< 0.01), predominantly in cortical neurons, and returned to baseline by 2 days. A delayed cerebral endothelial CCL2 message expression (*p *< 0.05) occurred 2 days after HPC. The levels of circulating monocytes (*p *< 0.0001), T lymphocytes (*p *< 0.0001), and granulocytes were decreased 12 h after HPC, and those of B lymphocytes were increased (*p *< 0.0001), but the magnitude of these respective changes did not differ between wild-type and CCL2-null mice. HPC did decrease the number of circulating CCR2^+ ^monocytes (*p *< 0.0001) in a CCL2-dependent manner, but immunohistochemical analyses at this 12 h timepoint indicated that this leukocyte subpopulation did not move into the CNS. While HPC reduced infarct volumes by 27% (*p *< 0.01) in wild-type mice, CCL2-null mice subjected to tMCAo were not protected by HPC. Moreover, administration of a CCL2 immunoneutralizing antibody prior to HPC completely blocked (*p *< 0.0001 vs. HPC-treated mice) the development of ischemic tolerance.

**Conclusions:**

The early expression of CCL2 in neurons, the delayed expression of CCL2 in cerebral endothelial cells, and CCL2-mediated actions on circulating CCR2^+ ^monocytes, appear to be required to establish ischemic tolerance to focal stroke in response to HPC, and thus represent a novel role for this chemokine in endogenous neurovascular protection.

## Background

Preconditioning occurs when an organism, tissue, or cell is exposed to a stressful, but non-damaging, stimulus that initiates genomic reprogramming for protection from subsequent injury [1-3]. One well-established model to induce "ischemic tolerance" in the central nervous system (CNS) utilizes a brief exposure to systemic hypoxia as the preconditioning stimulus (hypoxic preconditioning; HPC) to promote neurovascular protection in stroke [4-7]. Mechanistically, hypoxia activates survival-promoting signaling pathways responsible for altering gene expression via the upregulation of hypoxia-inducible factor-1 (HIF-1) [8]. HIF-1 modifies the expression of effector pathways that ultimately come to define the ischemia-tolerant phenotype, in part through the specific upregulation of HIF-1 in cortical neurons [8]. Following ischemia, HIF-1 mediated mechanisms contribute to cortical repair via the homing of progenitor cells to the site of injury [9], the upregulation of pro-angiogenic molecules [10,11], and the upregulation of erythropoietin [10,12].

CCL2, or monocyte chemoattractant protein (MCP)-1, is one of only two chemokines under the direct transcriptional control of HIF-1α regulation [13]. CCL2 is predominantly produced by astrocytes and resident microglia, and is traditionally known for its role in recruiting neutrophils and macrophages [14], as well as circulating neuroblasts [15], to sites of cortical injury under multiple pathological states. CCL2 is a full competitive agonist to its receptor, CCR2 [11], a Gαi-coupled receptor that modulates its signaling based on binding to individual CC-motif chemokines [12]. CCR2 is found on virtually all CNS cell types, including neurons, glial, endothelial, and immune cells [13-16], and is the only known receptor for CCL2 - although CCR2 also binds the chemokines CCL7, CCL8, CCL13, and CCL16 [16]. Several studies suggest a detrimental role for CCL2 in the progression of stroke injury, as both CCL2-/- [17] and CCR2-/- [18] mice exhibit reduced infarct volumes compared to wild-type controls.

Given its well-documented pro-inflammatory roles, CCL2 seems an unlikely candidate for inducing neuroprotection. However, since it is well established that harmful stimuli at higher doses can - at lower doses - serve as preconditioning stimuli, a role for chemokines in general, and CCL2 in particular, in the induction of ischemic tolerance is not necessarily unexpected. Indeed, several traditionally pro-inflammatory stimuli, including lipopolysaccharide (LPS) [18,19], tumor necrosis factor-α (TNF-α) [20], and even brief ischemia [21], upregulate signaling pathways that induce stroke tolerance. Evidence for the contribution of CCL2 to upstream cellular signaling during injury and repair shows that CCL2-CCR2 signaling upregulates transcription factors, including MCP-1-induced protein (MCPIP) [19] and Ets-1 [20], in monocytes and endothelial cells to initiate angiogenesis, a process that is critical to stroke recovery [21]. In addition, overexpression of CCL2 in cardiac myocytes protects during myocardial ischemia by activation of SAPK/JNK1/2 pathway [22], although, by implication, the activity of other signal transduction pathways downstream of CCR2 receptor activation (e.g., MAPK, ERK, and phospholipase C) may also participate in this epigenetic response [15,23,24].

Because of the direct upregulation of CCL2 by hypoxia and these signaling intermediary roles, we investigated whether CCL2 participates as a mediator of HPC-induced tolerance to stroke. We found that a single exposure to systemic hypoxia (our HPC stimulus) rapidly upregulates CCL2 mRNA and protein early in cortical neurons, with a delayed upregulation of CCL2 message in cortical microvessels. In the periphery, HPC reduced circulating granulocyte, T lymphocyte, and monocyte populations, while increasing B lymphocytes, in a CCL2-independent manner. However, CCL2 regulated the transmigration of CCR2^+ ^monocytes out of the peripheral blood in response to HPC. Moreover, in mice that lack bioavailable CCL2, either through genetic knockout or immunoneutralization, ischemic tolerance to HPC was not achieved, providing causal evidence for CCL2, likely produced by both neurons, cerebral endothelial cells, and circulating leukocytes, as a proximal signaling factor in HPC-induced gene induction pathways. While the fundamental mechanisms of CNS preconditioning have been under investigation for a couple of decades, this is the first evidence of chemokine signaling being critical to the induction of ischemic tolerance.

## Methods

### Hypoxic preconditioning (HPC)

The respective Institutional Animal Care and Use Committees at Washington University School of Medicine and University of Texas, Southwestern Medical Center approved all experimental procedures. Some experiments (i.e., those for CCL2 message and protein quantification, and immunohistochemistry) were carried out using SW/ND4 mice (Harlan Bioproducts) to match previously published results [5]. With the identification of CCL2 message and protein upregulation after HPC, remaining studies designed to assess causality were carried out in CCL2^-/-^/CX_3_CR1^GFP/+ ^mice on a C57BL/6 background (courtesy of Dr. Keiko Hirose, Washington University), with CCL2^+/+^/CX_3_CR1^GFP/+ ^and C57BL/6 wild-type controls. CX_3_CR1^GFP/+ ^mice have one functioning copy of the fractalkine receptor, CX_3_CR1, on monocytes, macrophages, and some dendritic/NK cells, which also fluoresce green. All studies used adult male mice, 25-35 g and 9-12 week old, randomized to experimental groups. Mice were preconditioned in modified home cages, with food and water available, and normobaric 8% O_2 _supplied continuously (1.5 L/min) for 4 h [5]. Outflow air was monitored via an oxygen analyzer (Vascular Technologies) to confirm the degree of ambient hypoxia. Naïve/control animals had no exposure to hypoxia.

### Quantitative rt-PCR

Animals were sacrificed 6 h through 2 week following hypoxic preconditioning (HPC). Following isoflurane overdose, animals were transcardially perfused with 20 mL 0.01 M PBS with heparin (1,000 units/mL) in RNAse-free sterile water. The neocortex was removed, and total RNA was isolated from cortical homogenates using standard techniques [25]. In the remaining hemisphere, a microvessel fraction (including largely capillaries, but also some small arterioles and venules) was isolated by differential centrifugation in sucrose buffer [5]. Primers (Integrated DNA Technologies, Coralville, IA) for CCL2 and CCR2 were normalized against copies of the housekeeping gene ribosomal 18S during quantitative real-time PCR (qPCR).

### Cerebral whole cell lysate immunoblotting

Animals were sacrificed as stated above for qPCR. Whole cell homogenates of perfused neocortices in lysis buffer were immunoblotted using standard protocols [7]. 85 μg of protein/well was loaded (10-20% gel; Bio-Rad, Hercules, CA), blocked, and incubated overnight in primary antibody solution (1:1000, CCL2 (Abcam, Cambridge, MA); 1:1000, CCR2 (Novus, Littleton, CO); 1:40,000, β-actin). Secondary antibodies (1:10,000; LiCor, Lincoln, NE) were image captured using the Li-Cor Odyssey Infrared Imaging System.

### Confocal immunofluorescent histochemistry

Animals were sacrificed 6 h through 2 week following HPC, at times corresponding to the quantitative rt-PCR analysis. Mice were transcardially perfused (20 mL 0.01 M PBS, 40 mL 4% paraformaldehyde/0.01 M PBS), brains cryoprotected in 30% sucrose, and sectioned at 10 μm in the coronal plane. Representative sections from the MCA territory were blocked and stained using standard procedures [5,25]. Primary antibodies detected CCL2 (1:20; PeproTech, Rocky Hill, NJ), neurons (NeuN 1:100; Millipore, Billerica, MA), astrocytes (GFAP 1:200; Molecular Probes, Grand Island, NY), or endothelial cells (CD31 1:50; BD Pharmingen, Franklin Lakes, NJ), followed by secondary antibodies (Alexa Fluor 488, 568, 598; 1:300; Invitrogen, Grand Island, NY) and counterstain (ToPro3; 1:300; Invitrogen). All photomicrograph images were obtained using an Olympus (Center Valley, PA) Fluoview (FV1000) confocal laser-scanning microscope or a Nanozoomer 2.0 (Hamamatsu, Bridgewater, NJ).

### Whole blood staining for flow cytometry

Blood was collected into EDTA-containing microtubes and stained to identify circulating leukocytes. After blocking Fc receptors with anti-CD16/CD32 (BD Biosciences, Billerica, MA), whole blood was stained with the following titrated antibodies (Ab): anti-CD45 APC to identify hematopoietic cells; anti-TCRβ PE-Cy5 to identify T lymphocytes; anti-CD4 PE-Texas Red to identify lymphocyte subsets; anti-Gr1 APC-Cy7 (BD Biosciences) to identify granulocytes and monocytes; anti-CD19 Alexa Fluor 700 to identify B lymphocytes; anti-CD11b Pacific Blue to identify monocytes (eBioscience, San Diego, CA); and anti-CCR2-PE (R&D Systems, Minneapolis, MN). Isotype-matched monoclonal antibodies were used to determine non-specific binding. Red blood cells were lysed using FACsLyse (BD Biosciences) according to the manufacturer's directions. Cells were immediately collected on the FACSAria (BD Biosciences) equipped with Diva Software. Data were analyzed using Flowjo software (Treestar, Ashland, OR).

### Transient focal cerebral ischemia

Mice were anesthetized using a brief exposure to 4% isoflurane/70% NO_2_/30% O_2_, with 1.8% isoflurane as a maintenance dose for the remainder of the procedure, as detailed previously [4,26,27]. In all mice, laser Doppler flowmetry (LDF; TSI, Inc., Shoreview, MN) measured relative change in cortical blood flow. Briefly, following topical preparation of the scalp, an incision at the temporal muscle exposed the left middle cerebral artery (MCA) territory, and the probe tip was targeted to the MCA territory based on anatomical landmarks. For transient middle cerebral artery occlusion (tMCAo), a ventral midline incision on the neck exposed the left common carotid artery, which was permanently ligated proximal to the suture placement. A silicon-coated, 6.0-gauge, nylon suture, 12 mm in length, was advanced 9.0-10.5 mm to transiently block the origin of the MCA, and confirmed with a second LDF reading, with > 80% reduction in relative blood flow to baseline required for inclusion. Body temperature was maintained at 37°C throughout the surgical procedure; animals were placed in a heated incubator (34°C) during ischemia. After either 35 or 45 min, animals were re-anesthetized, continued occlusion of the MCA confirmed by LDF, and the suture withdrawn. Successful reperfusion of the MCA territory was defined as a return of cortical blood flow > 50% of baseline at 10 min. Animals not meeting the above criteria were removed from the study.

### Infarct quantification

Animals were sacrificed 24 h following tMCAo by isoflurane overdose, then transcardially perfused with 20 mL heparinized saline. Upon removal of brains and gross examination, animals that underwent subarachnoid hemorrhage at the Circle of Willis, secondary to suture placement, were excluded from further analysis. Brains were sectioned on a 1.5-mm thick brain matrix and exposed to 2,3,5-triphenyl tetrazolium chloride (TTC) to delineate infarct regions. Infarct volumes were quantified by a blinded observer using standard image analysis software and corrected for edema based on corresponding right hemispheric areas as control [4,27].

### CCL2 immunoneutralization

Either control rat IgG IIb, or monoclonal CCL2 antibody (both from R&D Systems, Minneapolis, MN), were administered (2 mg/kg in sterile PBS, i.p.) 3 h before HPC [28].

### Statistical analyses

Data are presented as mean ± standard error of the mean (SEM), and the necessary group size confirmed by power analysis. Statistical comparisons were evaluated using student's *t*-test or one-way ANOVA, with Bonferroni post-hoc analysis (Prism, GraphPad, LaJolla, CA), for all experiments except the neurologic deficit scores, which were evaluated using Mann-Whitney rank sum test. Outliers outside the 95% confidence interval were excluded from final analysis, but are shown as open circles in the figures. Significance was determined as *p *< 0.05.

## Results and discussion

### Hypoxic preconditioning upregulates an early CCL2 response within the CNS

**Figure 1:**
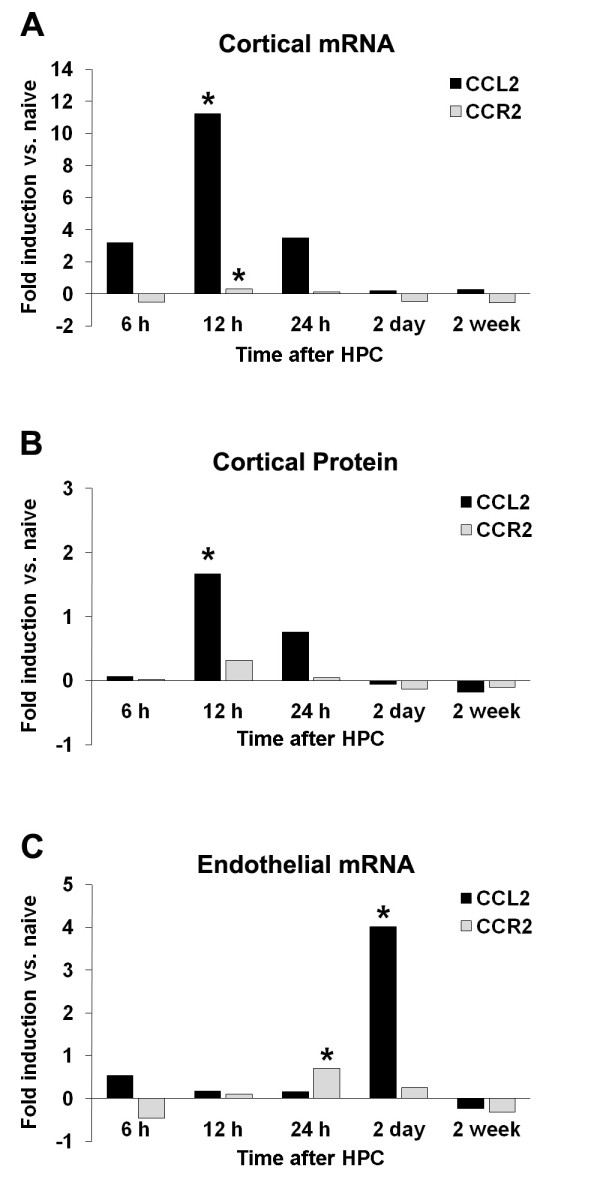
**Unique temporal profiles of hypoxic preconditioning induced upregulation of CCL2 message and protein in brain**. HPC (4 h, 8%O_2_) induces an early (A) 11-fold increase in cortical CCL2 mRNA and (B) 1.5-fold increase in CCL2 protein in cortical whole cell lysates, as well as a delayed (C) 3.5-fold increase in CCL2 mRNA expression in cortical endothelial cells (black bars) relative to naïve animals. CCR2 message and protein (grey bars) did not significantly change relative to naïve expression in cortical homogenates (A, B), but did in endothelial homogenates (C) within the same animals at 24 h after HPC. *p < 0.05 vs. respective expression level in naïves.

It is now appreciated that hypoxia stabilizes the alpha subunit of HIF-1, allowing it to dimerize to the beta subunit, translocate, and transcribe a host of both injurious and pro-survival genes in hypoxic cells, the relative expression of which depends on the severity and duration of hypoxic stimulus [29]. CCL2 contains a HIF-1 binding motif within its promoter region [13], and *in vitro *studies confirm hypoxia's ability to upregulate CCL2 in a HIF-1-dependent manner [13,30]. While *in vivo *systemic hypoxic exposure rapidly upregulates CCL2 expression in the periphery [31,32], whether brief hypoxia affects CCL2 within the CNS in a similar way was not known prior to our investigation. We therefore quantified CCL2 mRNA expression in hemispheric cortical homogenates from mice exposed to HPC 6 h through 2 week earlier (n = 7-8/group). Six hours after HPC, cortical CCL2 mRNA expression was increased 3-fold over naïve, and peaked at 11-fold over naïve 12 h after HPC (*p *< 0.01; Figure 1A). The elevation in cortical CCL2 message was fading by 24 h and was not distinguishable from baseline 2 days or 2 week after HPC. Our immunoblot analyses confirmed that cortical CCL2 protein expression also peaked at 12 h, with a 1.6-fold increase (*p *< 0.01) over naïve levels, and returned to baseline by 2 days following HPC (n = 4-7/group; Figure 1B). The expression change we observed, with a 12-h peak in CCL2 protein levels, was earlier than that occurring after permanent focal stroke [33], which suggests a potentially unique role for CCL2 following a mild, non-injurious hypoxic stimulus relative to its actions following complete ischemia. Since CCL2 is best known as a chemoattractant for monocytes/macrophages [15], we isolated microvessels from the remaining hemisphere of the same animals to quantify CCL2 mRNA expression in cerebral endothelium (Figure 1C). We found HPC-induced changes in cerebral endothelial cell CCL2 expression, but not temporally parallel with that we observed in cortical homogenates. Specifically, no changes in expression occurred between 6 h and 24 h after HPC, but a large 3.5-fold increase (*p *< 0.05) in expression was evidenced in the microvessel fraction 2 days after HPC. By 2 week post-HPC, expression levels were back to baseline. CCL2 expression within the vasculature is required for CCR2^+ ^monocyte rolling, adherence, and extravasation from blood into tissue beds [34,35]. Thus, this may reflect one aspect of the 'effector phenotype' established by the prior HPC stimulus, wherein the recruitment of monocytes to the brain could be enhanced at times more coincident with stroke onset (2 d), and not during the time of peak CCL2 expression (6-12 h), when CCL2 acts as a proximal signaling mediator for inducing adaptive responses in resident brain cells to protect them from a future ischemic event.

**Figure 2:**
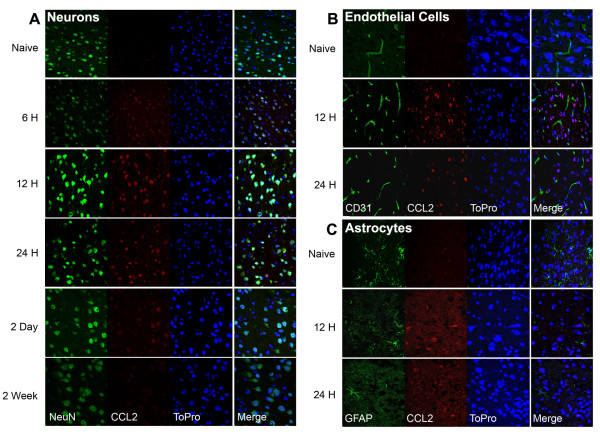
**CCL2 protein expression in brain after HPC**. (A) Temporal immunohistochemical analyses for CCL2 protein shows an early colocalization (at 6, 12, and 24 h) of CCL2 protein (red; middle panel) with NeuN-positive neurons (green; left panel) in the cortical grey matter after HPC (4 h, 8%O_2_). CCL2 reactivity was minimal in naïve animals and again at 2 days and 2 week after HPC. (B and C) Additional immunolabeling confirmed that CCL2 protein did not colocalize with (B) CD31-positive cortical endothelial cells (green; left panel), or with (C) GFAP-positive reactive astrocytes (green; left panel) within 24 h of HPC. ToPro3 (blue; right panel) was used in all photomicrographs as a general nuclear stain, and merged images are shown at the far right for each time point.

Ischemic injury *in vivo *upregulates CCL2 in several CNS cell types, including neurons [36,37], astrocytes [30,33,38,39], macrophages/microglia [33,39], and endothelial cells [37]. Because our qPCR analysis of the cortical microvascular fraction showed no upregulation of CCL2 message at 12 and 24 h after HPC, but our mRNA and protein measures in cortical homogenates revealed increased CCL2 expression at these times, we used a immunohistochemical approach to determine which remaining cell type contributed to the HPC-induced response. Figure 2 shows a temporal immunohistochemical analysis of HPC-induced CCL2 protein expression in the cortex, and our neuronal, astrocyte, and endothelial cell colocalization results. As with our qPCR and immunoblot findings, the strongest CCL2 immunoreactivity occurred 12 h and 24 h following HPC, and neurons were the predominant cell type expressing this chemokine. Regions with reactive, GFAP-positive astrocytes were rare, but typically found in cortical layer V/VI, near the corpus callosum, if present; nevertheless, there was minimal astrocyte expression of CCL2 in these regions during these times. A very small number of macrophages were also identified in the cortex at 6 h after HPC, and all of these colocalized with CCL2 (data not shown). Taken together, our results indicate that HPC induces a relatively rapid and highly neuron-specific upregulation of CCL2 within hours of the hypoxic exposure, and a delayed upregulation in cerebral endothelium two days after hypoxia, which suggested to us the hypothesis that this chemokine may contribute to the induction of the genomic reprogramming underpinning the ischemia-tolerant phenotype. Moreover, the finding of inactivation of HIF-1 transcription in neurons *in vitro *increases hypoxia-induced cell death, whereas the inactivation of HIF-1 in astrocytes in co-cultures is neuroprotective [40], is also consonant with our hypothesis that a brief, neuron-specific upregulation of CCL2, and perhaps other HIF-1 target genes, ultimately promote the ischemia-resistant phenotype.

At no time after HPC was the cortical expression of the CCL2 receptor, CCR2, significantly affected, although there were trends for both CCR2 mRNA (*p *= 0.06) and protein (*p *= 0.10) expression increases over naïve levels at 12 h following HPC (Figure 1A, B). In addition, there was no discernable qualitative difference in cortical CCR2 immunohistochemical expression following HPC (data not shown). There was a brief transient upregulation of endothelial CCR2 mRNA (*p *< 0.05) at 24 h after HPC that preceded the endothelial upregulation of CCL2 mRNA at 2 days. However, this response was not identified immunohistochemically at the protein level (Figure 1C). Although others have shown that, at least in monocytes, CCR2 message is downregulated following *in vitro *hypoxia [41], our *in vivo *findings indicate that HPC upregulates CCR2 within at least two resident brain cells, each along a distinct timeline. Even without a concomitant upregulation in CCR2 expression, the HPC-induced early upregulation in CCL2 production by neurons, and later in microvessels, is likely to have functional consequences, and implies CCL2-mediated autocrine/paracrine signaling is critical to the induction of ischemic tolerance.

### Hypoxic preconditioning alters circulating monocyte, granulocyte, and lymphocyte populations, but independent of CCL2

**Figure 3:**
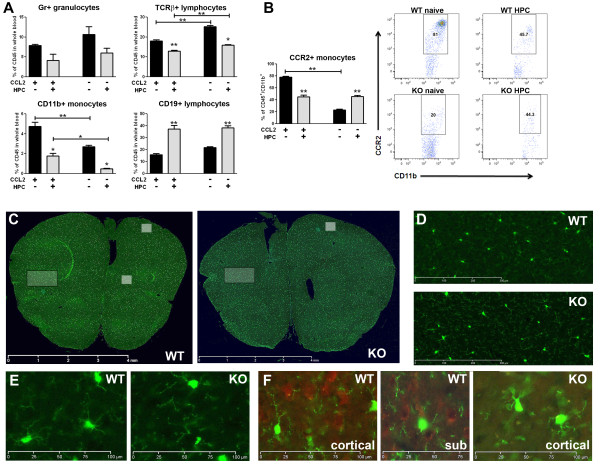
**HPC affects peripheral blood leukocyte populations**. (A) Whole blood staining identified baseline difference for TCRb^+ ^T lymphocytes and CD11b^+ ^monocytes in naïve (i.e., not exposed to hypoxia) CCL2 knockout (KO) mice versus naïve wild-type (WT) CX_3_CR1^GFP/+ ^mice. HPC reduced Gr-1^+ ^granulocytes, T lymphocytes, and monocytes, while increasing CD19+ B lymphocytes, to a similar extent in both strains. Data are shown as the mean percent of CD45^+ ^cells in the peripheral blood. (B) Representative staining for the chemokine receptor CCR2 on circulating monocytes showed significant strain differences at naïve levels that were equalized at 12 h after HPC. Whole blood was stained and expression of CCR2 by CD45^+^CD11b^+ ^monocytes was determined by comparison with monocytes stained with an isotype-matched control antibody. (C, D, E) Representative cortical sections from animals in the above experiment display a similar distribution pattern and morphology of resident CX_3_CR1-expressing monocytes/microglia at 12 h after HPC when seen at (D) 20 × and (E) 40 × magnification within the area denoted by the white rectangle. (F) CCL2 staining in adjacent sections, denoted by the white squares in cortical and subcortical areas. *p < 0.05, **p < 0.0001 vs. control.

Given that HPC is a systemic stimulus, hypoxia-induced changes in CCL2 expression in the periphery - particularly in circulating immune cells - may also contribute to the establishment of the ischemia-tolerant phenotype. We therefore examined whether HPC affected circulating white blood cell populations, and whether CCL2 modulates this response. Figure 3A shows the frequency of leukocyte subsets in whole blood, based on a CD45^+ ^gating [42], in both wild-type CX_3_CR1^GFP/+ ^and CCL2-null mice, under naïve conditions, and 12 h after HPC, the time when peak CCL2 protein expression occurred within the CNS. Under resting conditions, the genetic deletion of CCL2 (n = 7) resulted in higher basal levels of Gr1^+ ^granulocytes (26% increase) and B lymphocytes (28% increase) compared to wild-type CX_3_CR1^GFP/+ ^mice (n = 5), although the increases in each subpopulation were not statistically significant. TCRβ^+ ^T lymphocytes were similarly elevated in CCL2-null mice (28% over wild-type), and this increase was significant (*p *< 0.0001). In contrast, CD11b^+ ^monocytes were significantly decreased in CCL2-deficient animals (43% below wild-type; *p *< 0.001), which also resulted in a significant, 70% reduction in CD11b^+ ^monocytes expressing the CCL2 receptor CCR2^+ ^(*p *< 0.0001; Figure 3B). This previously reported monocytopenia in the blood of CCL2-null mice [43] reflects the loss of the highly specific CCL2/CCR2-mediated release of monocytes, but not neutrophils or lymphocytes, from the bone marrow of these animals [44,45].

In response to the mild systemic hypoxia of HPC, the peripheral distribution of leukocyte subsets was affected, but in a predominantly CCL2-independent manner. Specifically, HPC reduced TCRβ^+ ^T lymphocyte and CD11b^+ ^monocyte populations in both CX_3_CR1^GFP/+ ^wild type (n = 6; *p *< 0.0001 for both subsets) and CCL2-null (n = 5; *p *< 0.001 for both subsets) mice. While these populations are significantly different in HPC-treated CCL2-null mice compared to HPC-treated wild-types, the magnitude of reduction for both TCRβ^+ ^T lymphocytes (36% vs. 29%, respectively) and CD11b^+ ^monocytes (82% vs. 63%, respectively) was similar - a reflection of the different baseline expression levels found in naïve animals and not an altered response to HPC in the absence of CCL2. HPC induced a similar magnitude of downregulation of Gr1^+ ^granulocytes in both wild-type mice (47% reduction vs. naïve) and CCL2-null mice (44% reduction vs. naïve), and again the response did not differ between genotypes. B lymphocytes increased in response to HPC, with a 2.4-fold increase in CX_3_CR1^GFP/+ ^wild-types (*p *< 0.0001) and a 1.8-fold increase in CCL2-null mice (*p *< 0.0001) which, when normalized to all CD45^+ ^cells, was also unaffected by the presence or absence of CCL2 (37% vs. 38%, respectively).

### CCL2 mediates the movement of CCR2^+ ^monocytes from blood following hypoxic preconditioning

The only CCL2-dependent effect of HPC on the distribution of leukocyte subsets related to CCR2^+^/CD11b^+ ^monocytes. In wild-type mice, the 78% of monocytes that express CCR2 under resting conditions was reduced to 42% (*p *< 0.0001) following HPC, suggesting that about half of the CCR2^+ ^monocytes move out of the peripheral circulation in response to HPC. Conversely, in CCL2-null mice, HPC actually resulted in an increase in the fraction of CCR2^+ ^monocytes, from 23% to 49% (*p *< 0.0001), although this may reflect the movement of non-CCR2^+ ^monocytes out of the blood of CCL2-null mice, considering non-CCR2^+ ^monocytes constitute 77% of all monocytes in the knockouts. Coronal sections from the brains of the same mice used for the flow cytometry studies described above show a similar and evenly distributed resident monocyte population in both wild-type and CCL2-null mice at 12 h after HPC (Figure 3C-E). Given the well-known immune privilege of the CNS, which serves to limit leukocyte extravasation from the circulation into the brain under non-pathological conditions [25], we would contend that the mild hypoxic stimulus was not sufficient to increase monocyte extravasation across an intact blood-brain barrier in either wild-type or knockout mice. In fact, even during an increase in monocyte recruitment into the brain under bacterial pathogen-induced neuroinflammation, CCL2 gene deletion did not affect extravasation [46], due to either a CCR1-mediated chemotaxis [44,45,47], or the expression of CCL7 and CCL12 in CCL2-null mice [43]. Monocyte subpopulations extravasate into mesentery, lung, and skeletal muscle in response to mild hypoxia [48-50], which could account for their early movement out of the circulation in our study; future investigations should determine whether monocytes enter the brain at any later post-hypoxic timepoints. The delayed upregulation of CCL2 expression in cerebral endothelial cells 2 days after HPC would be consistent with such a possibility.

### Hypoxic preconditioning does not provide tolerance to transient focal stroke in CCL2-deficient mice

**Figure 4:**
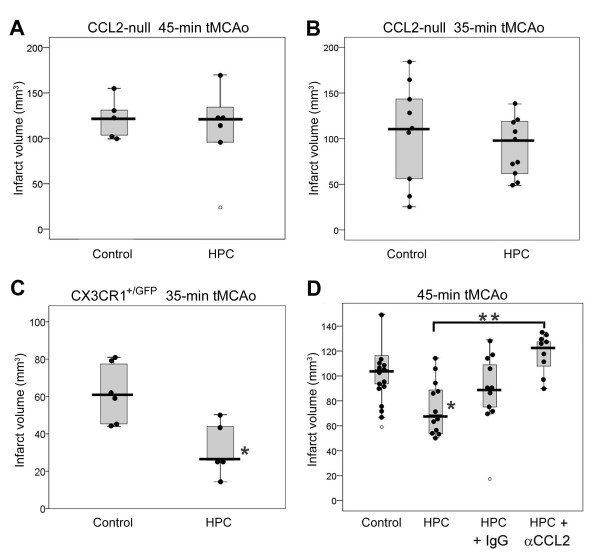
**Genetic and pharmacologic evidence for CCL2 involvement in HPC-induced stroke tolerance**. (A and B) HPC-induced ischemic tolerance was lacking in CCL2-null animals with HPC completed 2 days prior to (A) a 45-min tMCAo (n = 6) or (B) a 35-min tMCAo (n = 9) tMCAo, versus control CCL2-null mice with tMCAo but no prior HPC (n = 6 and n = 10, respectively). (C) HPC promoted ischemic tolerance to a 35-min tMCAo in CX_3_CR1^GFP/+ ^wild-type mice. (D) In WT mice, HPC 2 days prior to tMCAo (n = 12) reduced infarct volume resulting from 45-min of tMCAo versus controls with tMCAo but no prior HPC (n = 15). Immunoneutralization of CCL2 prior to HPC reversed this protection relative to untreated control mice with HPC (n = 9), and relative to control mice given the IgG-isotype control antibody prior to HPC (n = 11). Individual values (black-filled circles), mean (black horizontal bars), 75% confidence interval (gray box), and values (open circles) that fell outside of the 95% confidence interval (whiskers) are given. *p < 0.05, **p < 0.0001 vs. HPC.

Because the neuronal upregulation of HIF-1 is neuroprotective [40], and to test our hypothesis that HPC requires the HIF-mediated upregulation of neuronal CCL2 to induce the protective phenotype, we examined the ability of HPC to induce ischemic tolerance in CCL2-null mice following transient focal stroke [4]. Figure 4A shows 24 h post-stroke infarct volumes for non-preconditioned CCL2-null mice, and HPC-treated CCL2-null mice in response to a 45-min tMCAo. In the absence of CCL2, HPC exhibited no protective effect with respect to infarct volume (114 ± 16 mm^3^; n = 5) compared to non-preconditioned controls (114 ± 8 mm^3^; n = 6). These infarct volumes, however, are larger than those reported in prior studies of CCL2-/- mice following a 30-min tMCAo [51], and following permanent MCAo [17]. While infarct volumes in the latter study were similar to our previous experience with permanent MCAo [5], the smaller infarct volumes following tMCAo that others reported may be due to the use of different infarct quantification methods, as well as the shorter 30-min duration of ischemia. Schilling and colleagues [51] stained intermittent coronal sections with Toluidine Blue, and visually determined the infarct at the time of sectioning - which may have resulted in the exclusion of the most rostral and caudal portions of the infarct that are included using our TTC quantification protocol. We also examined another cohort of mice subjected to a 35-min tMCAo to ensure that the extent of damage caused by a 45-min occlusion did not mask or overwhelm any potential HPC-induced protection (Figure 4B). Again, HPC-treated CCL2-null mice had infarct volumes (87 ± 10 mm^3^; n = 10) nearly identical to non-preconditioned CCL2-nulls (91 ± 16 mm^3^; n = 9). Taken together, the results from these two cohorts confirm our hypothesis that CCL2 is required for HPC-induced tolerance to transient focal stroke.

Finally, recent studies show conflicting roles for the influence of CX_3_CR1 on the progression of ischemic injury, with genetic deletion of either CX_3_CL1 (i.e., fractalkine) or CX_3_CR1 decreasing post-stroke ischemic injury [52-54], whereas administration of CX_3_CL1 to wild-type rodents, with intact immune systems, promoting neuroprotection and long-term behavioral recovery [52]. Because our CCL2-null mice are on a CX_3_CR1^GFP/+ ^background, with one functional copy of the fractalkine receptor replaced by GFP in all CD11b^+ ^monocytes [55], we deemed it necessary to demonstrate that the single-copy deletion of CX_3_CR1 in the CCL2-null mice did not potentially mask cerebroprotection induced by HPC. However, as shown in Figure 4C, we confirmed that HPC prior to a 35-min tMCAo significantly reduced infarct volumes in CX_3_CR1^GFP/+ ^mice (32 ± 7 mm3; n = 5; *p *< 0.01) compared to non-preconditioned controls (62 ± 6; n = 6), indicating that the single-copy deletion of CX_3_CR1 had no effect on the ability of HPC to promote stroke tolerance.

### Immunoneutralization of CCL2 during hypoxic preconditioning blocks neuroprotection

If the HPC-induced neuronal and/or cerebral endothelial production of CCL2 acts as part of an autocrine/paracrine signaling mechanism, then preventing the CCL2-induced activation of CCR2 receptors should also inhibit HPC-induced tolerance in a manner similar to the effects of CCL2 gene deletion. To test this hypothesis, we immunoneutralized CCL2 during HPC in wild-type mice using a dose of monoclonal, anti-CCL2 neutralizing antibody that was shown previously to reduce infarct volumes in obese mice when administered intraperitoneally 3 h before stroke onset [28]. Figure 4D depicts infarct volumes 24 h following tMCAo in nonpreconditioned mice, and in preconditioned mice with and without the CCL2 neutralizing antibody. Note that, in untreated mice, HPC reduced infarct volume by 27% (75 ± 6 mm^3^; n = 12 vs. 102 ± 6 mm^3^; n = 15; *p *< 0.01). However, in mice receiving the antibody, the development of HPC-induced tolerance was blocked, resulting in infarct volumes (117 ± 5 mm^3^; n = 9; *p *= 0.16) significantly greater than HPC-treated mice with control IgG antibody (87.4 mm^3^; n = 11; *p *< 0.01), and 15% greater than nonpreconditioned controls. Our demonstration of the loss of the ischemia-tolerant phenotype by a CCL2-immunoneutralizing antibody, particularly when considered together with our aforementioned results in CCL2-null mice, serves as additional evidence implicating CCL2 in the HPC-induced triggering of the downstream signaling mechanisms that, in turn, are responsible for the changes in gene expression required for HPC-induced tolerance.

While our findings indicate that CCL2 is clearly implicated in the establishment of HPC-induced neuroprotection, it is unclear whether neuronal CCL2 mRNA and protein upregulation, cerebral endothelial cell CCL2 mRNA upregulation, and/or a peripheral recruitment of CCR2-expressing monocytes by CCL2, are required for triggering the genomic reprogramming responsible for ischemic tolerance to stroke. Although our findings implicate neurons as the predominant source of early CCL2 protein within the cortex following HPC, our histological findings would suggest they do not recruit from the circulation and into the CNS significant numbers of CCR2-expressing monocytes at the time of peak levels of CCL2 expression. In fact, in addition to monocytes, HPC appears to drive several leukocyte subpopulations out of the circulation, including granulocytes and T lymphocytes. Future flow cytometry experiments could determine (more definitively than immunohistochemistry) if any of these leukocyte subpopulations enter the CNS in response to HPC, and over what time periods after HPC. Perhaps the delayed upregulation of cerebral endothelial CCL2 that we observed two days after HPC helps promote CCR2^+ ^monocyte extravasation into the CNS at this later time, given that CCL2 expression is required for monocyte rolling and adherence to the venular endothelium [34,35]. This HPC-induced, CCL2-mediated recruitment of CCR2^+ ^monocytes from the periphery by cerebral endothelial cells would likely be blocked by a systemically-delivered CCL2 immunoneutralizing antibody, which could have contributed to the loss of HPC-induced stroke tolerance in mice receiving such treatment. Currently, few studies suggest any protective roles for monocytes in stroke injury and repair [56]. However, an elegant series of experiments following acute skeletal muscle injury show that CCL2 must be expressed by all three compartments - bone marrow, monocytes, and the injured skeletal muscle - to initiate phagocytosis of injured tissue and subsequent regeneration [43]. Thus, it is not inconceivable that the production of CCL2 from multiple cell types, and its subsequent autocrine, paracrine, and chemotactic actions, are needed to signal the induction of a pan-cellular ischemia-tolerant phenotype. A similar interplay between the neuronal-, endothelial-, and circulating immune cell-derived CCL2 and surrounding cells may also be foundational to the protective effects of "remote" preconditioning observed in both clinical and preclinical studies [57-60].

While hypoxia, via HIF-1, upregulates CCL2, it would be of interest to determine if the genomic reprogramming induced by other preconditioning stimuli also involves CCL2 signaling. Indeed, several well-described preconditioning stimuli, including LPS, TNF-α, interferon, and interleukin-1 beta (IL-1β), upregulate CCL2 [15]. Moreover, overexpression of CCL2 in cardiac myocytes mimics preconditioning through the upregulation of the phosphorylated SAPK/JNK1/2, but not ERK1/2 or p38, pathway [22], but whether these or similar signaling intermediates are involved in response to the more broad HPC stimulus is not presently known. The autocrine actions of neuronal CCL2 could also modulate post-stroke plasticity during recovery, given that exogenous CCL2 increases firing rates and excitatory postsynaptic transmission in CA1 hippocampal neurons [61], and/or influences neurotransmission - since CCR2 colocalizes with neurotransmitters in the cortex [62]. In addition to these novel, plasticity-based, neuronal functions, the paracrine effects of neuron- and cerebral endothelial cell-derived CCL2 on neighboring astrocytes, microglia, and other cells within the CNS, and on circulating immune cells, warrants further investigation.

## Conclusions

Stroke affects over 800,000 individuals per year in the United States, and although its rank as a cause of death has fallen to fourth, it remains the primary contributor to long-term adult disability [63]. Every effort should therefore be made to understand not only stroke pathology, but also any endogenous mechanisms that can be induced to establish a sustained ischemia-tolerant phenotype. Over a decade ago, we reported that a single exposure to systemic hypoxia, similar to levels tolerated by humans [64,65], imparted a period of tolerance to subsequent stroke injury [4]. In the present investigation, we showed that the neuronal and cerebral endothelial cell expression of CCL2 is upregulated in response to HPC. We also demonstrated a concomitant, predominantly CCL2-independent alteration in circulating leukocyte subpopulations by HPC. The spatio-temporal basis of these changes will require further refinement, particularly with regard to the potential for a delayed, endothelial cell-based, CCL2-mediated recruitment of monocytes into the CNS. Regardless, our findings that CCL2 gene deletion, or its immunoneutralization during HPC, robustly blocked HPC-induced stroke tolerance implicates CCL2 in its induction. Thus, this work advances a fundamentally new role for CCL2: Initiating the host of epigenetic changes in response to hypoxic preconditioning that ultimately establish a neurovascular-protective phenotype in the CNS.

## Abbreviations

Ab: antibodies; APC: antigen-presenting cell; CCL2: chemokine (C-C motif) ligand 2; CCR2: chemokine (C-C motif) receptor 2; CNS: central nervous system; CX_3_CL1: chemokine (C-X3-C motif) ligand 1; CX_3_CR1: CX3C chemokine receptor 1; D: day; EDTA: ethylenediaminetetraacetic acid; ERK: extracellular-signal-regulated kinase; GFAP: glial fibrillary acidic protein; GFP: green fluorescent protein; H: hour; HIF-1: hypoxia-inducible factor-1; HPC: hypoxic preconditioning; IgG: Immunoglobulin G; IL-1β: interleukin-1 beta; KO: knockout; LDF: laser Doppler flowmetry; MAPK: mitogen-activated protein kinase; MCA: middle cerebral artery; MCP-1: monocyte chemoattractant protein-1; mRNA: messenger ribonucleic acid; NK: natural killer; PBS: phosphate buffered saline; qPCR: quantitative real-time polymerase chain reaction; SAPK/JNK: stress-activated protein kinase/c-Jun NH2-terminal kinase; SEM: standard error of the mean; SW: Swiss-Webster; TCRβ: T cell receptor β; TTC: tetrazolium chloride; tMCAo: transient middle cerebral artery occlusion; TNF-α: tumor necrosis factor-α; WT: wild-type.

## Competing interests

The authors declare that they have no competing interests.

## Authors' contributions

AMS and JMG conceived of the study, designed the experiments, and co-wrote the manuscript. BKW conducted the CCL2 immunoneutralization studies, helped edit the manuscript, and made several other intellectual contributions to this work. ABF performed all of the rt-PCR and Western blot experiments, with the help of JLP. RH and JLP conducted the immunohistochemistry studies. AMS, PDC, ML, and OS designed and conducted the flow cytometry and immunohistochemical studies and aided in interpretation of data. All authors read and approved the final manuscript.
